# Ebbing Strength, Fading Power: Unveiling the Impact of Persistent Fatigue on Muscle Performance in COVID-19 Survivors

**DOI:** 10.3390/s24041250

**Published:** 2024-02-15

**Authors:** Mateusz Kowal, Ewa Morgiel, Sławomir Winiarski, Robert Dymarek, Weronika Bajer, Marta Madej, Agata Sebastian, Marcin Madziarski, Nicole Wedel, Krzysztof Proc, Katarzyna Madziarska, Piotr Wiland, Małgorzata Paprocka-Borowicz

**Affiliations:** 1 Department of Physiotherapy, Wroclaw Medical University, 50-368 Wroclaw, Poland; mateusz.kowal@umw.edu.pl (M.K.); weronika.bajer@umw.edu.pl (W.B.); malgorzata.paprocka-borowicz@umw.edu.pl (M.P.-B.); 2 Department of Rheumatology and Internal Medicine, Wroclaw Medical University, 50-367 Wroclaw, Poland; ewa.morgiel@umw.edu.pl (E.M.); marta.madej@umw.edu.pl (M.M.); agata.sebastian@umw.edu.pl (A.S.); piotr.wiland@umw.edu.pl (P.W.); 3 Biomechanics Department, Wroclaw University of Health and Sport Sciences, 51-612 Wroclaw, Poland; slawomir.winiarski@awf.wroc.pl; 4 Department of Rheumatology and Internal Medicine, University Teaching Hospital, 50-556 Wroclaw, Poland; madziarski.marcin@gmail.com (M.M.); lek.k.proc@gmail.com (K.P.); 5 Department of Medicine, Albert Einstein College of Medicine, Bronx, NY 10461, USA; nicole.wedel@einsteinmed.edu; 6 Clinical Department of Nephrology and Transplantation Medicine, Wroclaw Medical University, 50-556 Wroclaw, Poland; katarzyna.madziarska@umw.edu.pl

**Keywords:** isokinetic dynamometry, strength loss, fatigue, post-COVID-19

## Abstract

The total number of confirmed cases of COVID-19 caused by SARS-CoV-2 virus infection is over 621 million. Post-COVID-19 syndrome, also known as long COVID or long-haul COVID, refers to a persistent condition where individuals experience symptoms and health issues after the acute phase of COVID-19. The aim of this study was to assess the strength and fatigue of skeletal muscles in people recovered from COVID-19. A total of 94 individuals took part in this cross-sectional study, with 45 participants (referred to as the Post-COVID Cohort, PCC) and 49 healthy age-matched volunteers (Healthy Control Cohort, HCC). This research article uses the direct dynamometry method to provide a detailed analysis of post-COVID survivors’ strength and power characteristics. The Biodex System 4 Pro was utilized to evaluate muscle strength characteristics during the fatigue test. The fatigue work in extensors and flexors was significantly higher in the PCC. The PCC also showed significantly less power in both extensors and flexors compared to the HCC. In conclusion, this study provides compelling evidence of the impact of post-COVID-19 fatigue on muscle performance, highlighting the importance of considering these effects in the rehabilitation and care of individuals recovering from the virus. PCC achieved lower muscle strength values than HCC.

## 1. Introduction

Post-COVID-19 syndrome, also known as long COVID or long-haul COVID, refers to a persistent condition where individuals experience symptoms and health issues after the acute phase of COVID-19. The symptoms can vary from person to person, but some commonly reported ones include fatigue or extreme tiredness [1,2,3,4], shortness of breath or difficulty breathing, cognitive difficulties (brain fog) [5,6], muscle or joint or chest pain or tightness [7,8,9], different headaches [10], loss of taste or smell [11,12,13], sleep disturbances or insomnia [14,15], palpitations or heart racing, and others, like gastrointestinal issues such as nausea, diarrhoea, or abdominal pain, depression or anxiety, post-exertional malaise (worsening of symptoms after physical or mental exertion), dizziness or light-headedness. The prevalence of long COVID syndrome is difficult to determine unequivocally, mainly due to the different definitions used and methods of observation. Post-infectious symptoms are variable in severity and involve different systems, and previous observations suggest that the incidence of long COVID may depend on the population studied, and factors such as sex, age, severity of infection, and viral mutation. Among the most commonly reported post-COVID symptoms are many related to physical performance and capacity, e.g., fatigue, which in some observations was reported by up to 80% of patients. In another observation, almost 40% of COVID patients were unable to return to their daily activities 60 days after discharge from hospital [16]. In another study of 219 hospitalised patients, performance impairment was confirmed by activity tests in 59% of the patients [17]. It is important to note that the symptoms and their severity may vary widely among individuals. Some people may experience only a few, while others may have a broader range of symptoms. The duration of symptoms also varies, with some individuals experiencing them for weeks or months [18,19,20].

Persisting muscle fatigue can have a significant influence on daily-living human performance, i.e., muscle fatigue reduces the ability of muscles to generate force, leading to decreased muscle strength and power output [21,22], which can impair performance in activities requiring strength, such as weightlifting, running, or stair climbing; it can limit the ability to sustain prolonged physical activity or exercise leading to decreased endurance performance [23], or may exhibit diminished motor control and coordination, resulting in compromised movement precision and accuracy [24].

Furthermore, as fatigue sets in, individuals may exhibit altered movement patterns and compensatory strategies to overcome muscle weakness [25]. These changes in mechanics can negatively affect performance efficiency and increase the risk of injury or slow down reaction times, impairing performance in tasks requiring quick decision making and response speed [26]. Moreover, muscle fatigue can also indirectly affect cognitive function, including decreased attention, concentration, and decision-making abilities [27]. This can impact performance in mentally demanding tasks that require focus and precision. This subjective increase in fatigue perception can negatively influence motivation and psychological well-being, affecting overall performance [28].

The knee joint provides stability, generates force, and controls locomotion. Knee muscle strength and power are crucial for performing various functional movements involved in daily living activities, such as walking, climbing stairs, squatting, and rising from a seated position [29]. These movements rely heavily on the ability of knee muscles to generate force and control joint motion [30]. Strong knee extensor muscles, such as the quadriceps, are particularly important for activities that involve pushing off or lifting the body. Adequate knee muscle strength is also essential for maintaining joint stability and preventing excessive motion or instability at the knee. Strong knee muscles, including the quadriceps and hamstrings, help stabilise the knee joint during weight-bearing activities and provide support to prevent injuries by absorbing and dissipating forces during physical activities [31].

This research study examines the relationship between persistent fatigue (or extreme tiredness) symptoms in individuals who have recovered from COVID-19 and its impact on muscle strength and power during muscle contraction. This study seeks to uncover the hidden effects of post-COVID-19 fatigue on physical performance, providing valuable insights into the potential implications for individuals’ overall physical functioning. We hypothesised that individuals who were hospitalised with COVID-19 would have less strength and muscle power than individuals in the age-matched, healthy control group.

## 2. Materials and Methods

### 2.1. Study Group

A total of 94 individuals took part in this cross-sectional study, with 45 participants (referred to as the Post-COVID Cohort, PCC) selected from patients who were hospitalised in the internal medicine department of the University Teaching Hospital between 1 February 2020 and 31 December 2022. The selection criteria for the PCC required a confirmed diagnosis of COVID-19 through PCR testing. Inclusion criteria for hospital admission were age ≥ 18 years and the need for oxygen therapy or pharmacological treatment during hospitalisation (e.g., IV glucocorticosteroids, IV antibiotics, remdesivir, baricitinib, blood product transfusion). Patients requiring mechanical ventilation and intensive care unit (ICU) care at admission were excluded. A retrospective analysis of hospitalisation data identified patients aged 20–50 without chronic diseases that could potentially impact the study results, such as a history of vascular incidents (e.g., stroke, myocardial infarction, thromboembolism), chronic circulatory failure, chronic respiratory failure, or chronic musculoskeletal diseases. Patients with medical contraindications to exercise were also excluded. Phone calls were made to identify individuals willing to participate in the study, and informed consent was obtained from all participants. The Healthy Control Cohort (HCC) consisted of 49 healthy age-matched volunteers. Both study groups met WHO recommendations for physical activity. Table 1 presents the characteristics of the study groups.

### 2.2. Experimental Set-Up

**Figure 1:**
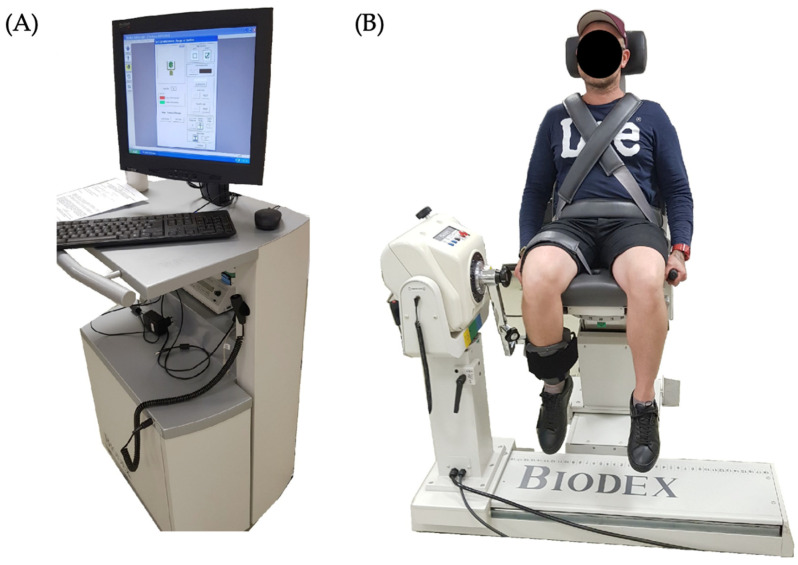
Biodex System 4 Pro (**A**) and participant’s positioning during test (**B**).

The multifunctional device Biodex System 4 Pro (Biodex Medical Systems Inc., Shirley, NY, USA) was utilised to assess knee muscle strength and power. The system consists of an electrically adjustable dynamometer. This electronically adjustable rotational and sliding chair allows rotational and sliding movements. The mobile workstation is equipped with a control panel for data analysis, archiving, and exporting. The speed control mechanism regulates the angular velocity of the movement, with the protocol set at 60 degrees per second for this study. Additionally, the system incorporates a resistance mechanism that provides adjustable resistance to match the patient’s strength level. The participants were positioned comfortably in the Biodex System 4 Pro, with their knee joints aligned with the dynamometer’s axis. Proper stabilisation and alignment of the body were ensured to minimise extraneous movements (Figure 1).

The strength of knee flexors and extensors was assessed under static and dynamic contractions. For measuring isokinetic knee flexion/extension, the following steps were followed: (1) The patient was seated on the Biodex System 4 Pro, with the knee joint aligned with the dynamometer’s axis. The patient’s forefoot was securely attached to the lever arm of the device. (2) Before testing began, a warm-up session was typically conducted to prepare the patient’s muscles and joints for the isokinetic exercise. This involved light stretching, range of motion exercises and low-resistance repetitions. (3) The technician selected the specific measurement range of motion (customising the start and end angles for the movement) for the knee flexion/extension exercise. This could vary depending on the patient’s joint mobility condition. (4) The technician set the desired speed to 60 degrees per second, matching the speeds of daily living activities. (5) Before the data collection started, a few familiarisation trials were conducted to ensure the patient understood the movement and became comfortable with the device. This helped minimise learning effects and improved the accuracy of subsequent measurements. (6) The actual isokinetic knee flexion/extension testing started once the patient was ready. The Biodex System 4 Pro recorded the torque (moment of force) exerted by the patient’s muscles, the angular velocity (speed) of the knee movement, and other relevant parameters in real time. These data were collected throughout the specified range of motion and at the predetermined speed. (7) The testing protocol involved four trials of 30 repetitions of knee flexion/extension movements. Between each set, a rest period of 1 min was provided to allow the patient to recover and minimise the effects of fatigue. (8) After the completion of the testing session, the collected data were analysed and interpreted.

The Biodex System 4 Pro software allowed us to assess the following parameters for both flexion and extension movements:

1. PKTQ/BW—the peak torque (in newton metres, Nm) normalised by body weight (kilograms of force, kG) expressed as a percentage of the participant’s body weight. It provides a relative measure of muscle strength.
2. TIME TO PKTQ—the duration it takes for the patient to reach the peak torque value during the exercise. It is measured in milliseconds (ms). A shorter “TIME TO PKTQ” may indicate efficient neuromuscular coordination and faster muscle activation, suggesting better overall muscle performance. On the other hand, a longer “TIME TO PKTQ” may suggest delayed muscle activation or neuromuscular coordination, potentially indicating suboptimal muscle function or potential muscular fatigue [32].
3. ANGLE OF PKTQ—the joint angle (in degrees) at which the peak torque is achieved. It indicates the specific position of the joint where the maximum muscle force occurs. The angle at which peak torque is reached signifies the optimal alignment or positioning of the joint for generating maximum force. This angle reflects the biomechanical advantage of the muscle in generating torque, as it represents the joint configuration that allows for optimal muscle length-tension relationship and leverage. A joint angle closer to the “ANGLE OF PKTQ” suggests that the muscle can generate maximal force in that specific position. Deviations from this optimal angle may decrease force production due to suboptimal muscle length or altered leverage [33].
4. TQ@30DEG—the muscle’s measured torque when the joint is at a 30-degree angle. It is measured in newton metres (Nm). The choice of 30 degrees is used as a standardised angle in isokinetic testing protocols, particularly for knee flexion-extension measurements. This degree represents a significant portion of the joint’s range of motion, allowing for meaningful muscle strength and performance assessment. It is often considered a mid-range position commonly used in clinical and research settings [33].
5. COV—a statistical measure that represents the variability/dispersion of the measured values or curves. It is calculated for the whole torque-angle curve as the standard deviation divided by the mean and expressed as a percentage. A higher COV indicates a more significant relative dispersion or variability (reliability and stability) of the measurement. A lower COV suggests a more homogeneous or consistent dataset, suggesting less consistency or more diverse responses among participants.
6. WRK/BW—the average total work normalised by body weight expressed as a percentage of the participant’s body weight. It provides a relative measure of the amount of work performed. A higher value indicates a more significant amount of work relative to body weight and suggests increased muscular effort and energy expenditure. Normalising the total work to body weight allows for comparisons between individuals of different body sizes and provides insights into the efficiency and effort exerted during the exercise [32].
7. WORK FIRST THIRD—the average work value during the initial one-third portion of the exercise (first ten repetitions) in each trial, typically calculated using the first ten repetitions. It is measured in joules (J). It provides insight into the energy expenditure and muscular effort during the initial phase of the exercise [34].
8. WORK LAST THIRD—the average work value during the final one-third portion of the exercise, calculated using the last ten repetitions. It is measured in joules (J). This variable can provide information about fatigue levels, changes in muscle performance, and the ability to sustain effort towards the end of the movement pattern [34].
9. WORK FATIGUE—the ratio of work in the last third to work in the first third (reduced by 100%) and expressed as a percentage. It indicates muscle fatigue levels (% of strength loss) experienced during the exercise. A higher percentage value suggests a more significant decline in work output towards the end of the exercise, indicating increased muscle fatigue. By comparing the “WORK FIRST THIRD” and “WORK LAST THIRD” values, one can gain insights into the energy distribution and potential changes in muscular performance throughout the exercise. These variables contribute to a comprehensive understanding of muscle function, fatigue patterns, and the ability to maintain work output over time [32].
10. AVG POWER—the average rate at which work is performed, representing the speed of work execution. It is measured in watts (W). A higher AVG POWER value indicates a faster rate of work execution, suggesting greater muscular strength and efficiency in generating force [32].
11. AGON/ANTAG RATIO—the agonist-to-antagonist muscle ratio expressed as a percentage. During the exercise, it indicates the relative contribution or balance between the primary muscle group (agonist) and the opposing muscle group (antagonist). A higher ratio (>1) suggests a stronger contribution from the agonist muscles, and a lower ratio (<1) suggests a stronger contribution from the antagonist [35].

The experimental set-up utilized the Biodex System 4 Pro for the assessment of knee muscle strength and power. This system’s validity and reliability are well-established in the literature. Specifically, intra-class correlations (ICCs) for knee flexion and extension were found to be good, ranging from 0.79 to 0.89, at a velocity of 60°/s [32]. Furthermore, another study investigating knee extensor strength in healthy adults reported excellent intra- and inter-rater reliability of observed mean peak torque values with an ICC of 0.85 for maximum measurements of knee extensor strength [36]. These findings collectively affirm the Biodex System 4 Pro’s capability in providing reliable and valid assessments of muscle strength, thereby supporting its use in our study.

### 2.3. Ethical Considerations

Ethical approval was obtained from the Bioethics Committee at Wroclaw Medical University (approval no. KB-1080/2021), and written informed consent was obtained from all participants prior to their inclusion in the study.

### 2.4. Statistical Analysis

All statistical analyses were performed using the Statistica (STATISTICA 13.1) software. The level of significance in all tests was set at *p* ≤ 0.05. Descriptive statistics were calculated to summarise the characteristics of the study participants, including mean values and standard deviations. Assumptions of the statistical analyses, such as normality of the residuals, homogeneity of variances, and absence of multicollinearity, were checked and met as appropriate. The normality of the distribution was tested using the Shapiro–Wilk test (with a null hypothesis that the data are derived from a normally distributed population with a *p*-value greater than the chosen significance level). A commonly used Levene’s test was used to check the homogeneity of variances. To check the absence of multicollinearity between measurement parameters in the PCC and HCC, we employed a variance inflation factor (VIF): VIF = 1/(1 − R^2^), where R^2^ is the coefficient of determination from the regression model for each independent variable. The VIF assesses the correlation between each independent variable (measurement parameter) and other independent variables in the multiple regression model with the measurement parameters as independent variables. VIF values below the threshold of 10 were considered insignificant multicollinearity issues [37].

The independent samples *t*-test for means in independent groups was used to compare the PCC and HCC. Correlation analyses examined the association between persistent fatigue symptoms and muscle strength/power. The Pearson correlation coefficients were calculated to determine the strength and direction of the linear relationship between the variables. In addition, Cohen’s d was calculated to indicate the standardised difference between group means to assess the effect size. Cohen’s d = (M1 − M2)/SDpooled, and SDpooled = sqrt[((n1 − 1)SD1^2^ + (n2 − 1)SD2^2^)/(n1 + n2 − 2)], where SDpooled is the pooled standard deviation, n1 and n2 are the sample sizes of the PCC and HCC, SD1 and SD2 are the standard deviations of the cohorts. A Cohen’s d < 0.2 was considered a “small” effect size, 0.2 < d < 0.5 a “medium” effect size, and d > 0.8 a “large” effect size.

All statistical tests were two-tailed, and a significance level of *p* < 0.05 was considered statistically significant. Effect sizes were reported where appropriate to estimate the practical significance of the observed relationships.

## 3. Results

In comparing the Post-COVID Cohort (PCC) and the Healthy Control Cohort (HCC), there were significant differences in numerous knee extensor and flexor performance measures, with large effect sizes evident in most parameters (Table 2).

The PCC demonstrated significantly lower relative peak torque to body weight (PKTQ_BW) for both extensors and flexors compared to the HCC with large effect sizes (Ext: d = 2.1, Flx: d = 1.3). The t-tests revealed significant differences (*p* < 0.001) for both variables, with the difference of means for Ext and Flx falling in the range of −45.5 to −30.3 and −22.0 to −11.5, respectively. Regarding the time to reach peak torque, there was a significant difference (*p* = 0.02) for the extensors, with the PCC taking longer (672.7 ± 54.6 ms) compared to the HCC (638.9 ± 80.8 ms), with a small effect size (d = 0.5). However, the flexors had no significant difference (*p* = 0.65), with a negligible effect size (d = 0.1).

The PCC also demonstrated significantly different angles at peak knee torque for both extensors and flexors compared to the HCC, with large effect sizes. The PCC had a smaller angle for extensors (67.8 ± 4.0°) and a larger angle for flexors (62.4 ± 6.0°) compared to the HCC. The t-tests revealed significant differences (*p* < 0.001) for both variables. Regarding torque at 30 degrees, the PCC demonstrated significantly lower values for both extensors (89.8 ± 24.7 Nm) and flexors (40.3 ± 13.8 Nm) compared to the HCC with medium to large effect sizes (Ext: d = 0.7, Flx: d = 0.9). The t-tests revealed significant differences (*p* < 0.001) for both variables. Furthermore, in the coefficient of variation of torque, the PCC demonstrated a significantly higher value for extensors (26.2 ± 8.1%) compared to the HCC (17.3 ± 4.1%) with a large effect size (d = 1.4). For the relative work to body weight, the PCC demonstrated significantly lower values for both extensors (233.1 ± 24.6%) and flexors (102.4 ± 15.1%) compared to the HCC (Ext: 263.5 ± 23.6%, Flx: 127.6 ± 14.1%) with large effect sizes (Ext: d = 1.3, Flx: d = 1.7). In terms of work done in the initial third of the trial, both extensors and flexors in the PCC demonstrated significantly lesser values compared to the HCC. Significant differences were observed for the work done in the terminal third of the motion, with the PCC showing less work output.

Also, significant differences were noted in the evaluation of work fatigue and power between the Post-COVID Cohort (PCC) and the Healthy Control Cohort (HCC). For the extensors (Ext) and flexors (Flx) of the knee regarding work fatigue, the PCC showed significantly higher values than the HCC. The PCC exhibited work fatigue of 37.7 ± 21.1 J for the extensors and 56.8 ± 23.5 J for the flexors. In comparison, the HCC reported 18.9 ± 13 J for the extensors and 39.0 ± 11.8 J for the flexors. The *t*-test for work fatigue in extensors and flexors showed *t*-statistics of 5.1 (*p* < 0.001, 95% CI [11.5, 26.1], Cohen’s d = 1.1) and 4.6 (*p* < 0.001, 95% CI [10.1, 25.6], Cohen’s d = 1.0), respectively, suggesting significantly larger work fatigue in the PCC.

Regarding muscle power, the PCC exhibited lower power than the HCC for both extensors and flexors. The power values for the PCC were 98.4 ± 12.0 W for the extensors and 39.9 ± 9.2 W for the flexors, while the HCC exhibited 103.9 ± 8.3 W for the extensors and 50.2 ± 8.4 W for the flexors. The t-tests for power in extensors and flexors showed *t*-statistics of −2.5 (*p* = 0.01, 95% CI [−9.8, −1.1], Cohen’s d = 0.5) and −5.5 (*p* < 0.001, 95% CI [−14.0, −6.6], Cohen’s d = 1.2), respectively, indicating a significant decrease in power in the PCC.

The fatigue work in extensors and flexors was significantly higher in the PCC. The PCC also showed significantly less power in both extensors and flexors compared to the HCC.

## 4. Discussion

In summary, these results suggest that individuals in the PCC experience greater work fatigue and reduced power in the knee extensors and flexors compared to those in the HCC. In contrast, no significant difference was observed in the agonist/antagonist ratio. The results suggest that post-COVID patients may exhibit lower muscle strength and work capacity and greater fatigue compared to healthy controls. The effect sizes for these differences, as measured by Cohen’s d, were significant for most variables.

Moreover, this research provides evidence of the substantial effect of post-COVID-19 fatigue on the muscle strength and power of patients who have recovered from the virus. Significant disparities between the Post-COVID Cohort (PCC) and the Healthy Control Cohort (HCC) were observed across multiple measures of muscle performance. Lower relative peak torque to body weight, longer time to reach peak torque for extensors, differing angles at peak knee torque, lower torque at 30 degrees, higher coefficient of variation of torque for extensors, lower relative work to body weight, and lesser work done in the initial and terminal thirds of the trial were all detected in the PCC. Furthermore, the PCC demonstrated increased work fatigue and decreased muscle power compared to the HCC. However, no significant difference was observed in the agonist/antagonist ratio. The most noteworthy finding was the lower relative peak torque to body weight in the PCC compared to the HCC, with large effect sizes. This suggests that individuals in the PCC had significantly weaker knee extensors and flexors, which might directly affect their overall physical performance, mobility, and quality of life.

The finding that the PCC took longer to reach peak torque for extensors might imply that their muscles cannot generate force rapidly, which could indicate impaired muscle power. Furthermore, the observation of differing angles at peak knee torque in the PCC compared to the HCC suggests alterations in the length-tension relationship of the muscles. This could impact the biomechanics of movements, such as walking or running. Interestingly, the study found no significant difference in the agonist/antagonist ratio between the PCC and HCC. This might suggest that post-COVID fatigue does not disproportionately affect the muscles involved in knee extension and flexion. However, the implications of this observation warrant further exploration.

The findings of increased work fatigue and decreased muscle power in the PCC underscore post-COVID fatigue’s persistent and debilitating effects. This could have serious implications for the daily functioning of individuals in the PCC, who might struggle with tasks that require sustained muscle contraction or rapid force generation.

In terms of the broader research landscape, one study found that patients with long COVID had significantly lower absolute and relative muscle strength measurements than control participants, supporting the adverse effects of long COVID on muscle function. The relationship between long COVID and grip/leg strength levels was partly mediated by limb muscle mass, suggesting that the evident reduced upper and lower muscle mass might be a potential cause or contributor to the functional limitation of patients with long COVID [38].

However, more research is needed to understand the underlying mechanisms of these findings. Investigating the potential role of factors such as inflammation, metabolic changes, and neurological effects in contributing to these muscle performance disparities would be valuable. Furthermore, future studies could also explore potential interventions to mitigate these effects, such as physical therapy or nutritional supplementation. Also, longitudinal studies tracking the progression of these effects over time would provide further insights. Our study’s findings regarding the impact of COVID-19 on muscle strength and performance align with and extend upon the existing literature in this domain. Previous studies have indicated a marked decline in musculoskeletal functionality in post-COVID-19 patients, highlighting prolonged recovery periods and the need for targeted rehabilitation strategies. A pivotal study by Chandra et al. [39], outlined the direct and indirect mechanisms through which SARS-CoV-2 affects skeletal muscle cells and the inflammatory responses triggered by the virus. This aligns with our observation of strength loss in COVID-19 survivors, suggesting a potential link between these molecular mechanisms and the clinical manifestations of muscle weakness and fatigue. Furthermore, our findings contribute to the broader discourse on COVID-19′s musculoskeletal impact, as reported by Awosanya et al. [40], which emphasizes the multifaceted nature of the virus’s effects on musculoskeletal health, including bone loss and altered osteoclastogenesis. These insights collectively underscore the need for a comprehensive evaluation of COVID-19 survivors, considering both molecular and functional aspects.

The isokinetic dynamometry device, such as the Biodex System, is considered a gold standard in muscle strength testing, and its test–retest reliability has been confirmed to be excellent (with ICC ranging from 0.94 to 0.98 for knee extension and flexion) in physically active adults [34]. It is also a standard used in rehabilitation to assess the muscle strength of people after anterior cruciate ligament reconstruction (predominance of extensors over flexors), and to assess the risk of injury (proportion between flexors and extensors) [41,42]. The optimal angle at peak torque for extensors is believed to be 70–85 degrees, and it is 20–30 degrees for flexors in isometric conditions. The optimal angle at peak torque changes with speed. For example, in a study assessing the relationship between isokinetic peak torque and angle-specific torques in the knee, it was found that at low (60°/s) and moderate (180°/s) speeds, the knee angle of hamstrings and quadriceps peak torque usually occurs between 30 and 60 degrees of knee flexion. Another study found that at 60°/s, the mean peak torque for the hamstrings occurred at 33 degrees for men and 37 degrees for women [43]. However, it is essential to note that these values can vary depending on factors such as muscle strength, injury type, and the specific joint being tested. According to Kannus and Yasuda [44], the mean peak torque angle for the hamstrings was 35 degrees of knee flexion when the angular speed of the dynamometer was 60°/s. The average peak torque angle for the quadriceps was 54 degrees at 60°/s. These values were obtained using a Cybex II dynamometer to measure isokinetic peak torques at slow (60°/s) and moderate (180°/s) speeds in patients with different knee ligament insufficiencies.

Similarly, there are some normative values for torque at different angles for flexors and extensors. For example, Kannus and Jarvinen found that at 60°/s, the mean peak torque for the hamstrings occurred at 33 degrees for men and 37 degrees for women [45]. Moreover, Pinter et al. found that for male participants, the mean maximal torque was 85 (±6) Nm for flexors and 77 (±9) Nm for extensors, while for female participants, the values were 48 (±3) Nm for flexors and 36 (±2) Nm for extensors [46].

Generally, in a healthy population, the knee extensors (quadriceps) are typically stronger and generate more torque than the knee flexors (hamstrings). This is primarily due to the anatomical structure and function of the muscles involved. The quadriceps muscle group, which includes the rectus femoris, vastus lateralis, vastus medialis, and vastus intermedius, is responsible for extending or straightening the knee. These muscles are larger and generally more powerful than the hamstrings. Agonist-to-antagonist strength ratios, such as the hamstring-to-quadriceps (H:Q) ratio, are often used in sports medicine and physical therapy to assess muscle balance and injury risk. An imbalanced H:Q ratio, where the strength of the hamstrings is significantly lesser than that of the quadriceps, can increase the risk of injuries such as hamstring strains and anterior cruciate ligament (ACL) tears. Normative values for the hamstring-to-quadriceps (H:Q) ratio can vary depending on the population and the specific testing protocol used. However, a commonly cited normative value for the conventional (or concentric) H:Q ratio in a healthy adult population is around 0.6, meaning the hamstrings are about 60% as strong as the quadriceps. However, it is generally understood that the H:Q ratio can vary with the speed of muscle contractions during isokinetic testing. Typically, the H:Q ratio decreases as the contraction speed increases. This is mainly because the quadriceps (particularly the fast-twitch muscle fibres) can generate more force at higher speeds than the hamstrings. According to Hevett et al., males demonstrated a significant correlation between H:Q ratio and isokinetic velocity (R = 0.634, *p* < 0.0001) and a significant difference in the isokinetic H:Q ratio at the lowest angular velocity (47.8 ± 2.2% at 30°/s) compared to the highest velocity (81.4 ± 1.1% at 360°/s, *p* < 0.001) [47]. This means that in males, the H:Q ratio increases with increasing isokinetic velocity during testing, reaching 60 ± 5% for men and 53 ± 6% for women [47]. While the specific values can vary based on numerous factors (including age, sex, and training status), normative H:Q ratios at slow speeds (like 60°/s) are often around 0.6–0.75 and can decrease to around 0.5 or lower at faster speeds (180–240°/s). A study on football players’ functional hamstring to quadriceps ratio found that H:Q ratios were 0.9, 1.03, and 1.04, respectively, for isokinetic measurements with 60°/s [48]. Another study on professional male soccer players found that the H:Q conventional ratio mean scores were close to 60% when tested at low to intermediate angular velocities and around 70–80% at fast angular velocities. The H:Q functional ratio mean scores were close to 80% at 60°/s, around 100–130% at intermediate to fast angular velocities, and near or above 130% when angular testing velocities were mixed [48].

Similarly, work fatigue (or strength loss) is seldom reported in the literature. Generally, it is recognised in the literature that work fatigue can significantly affect the performance of knee flexors and extensors. Fatigue can result in a decrease in muscle power output, which could potentially affect the work produced by these muscles. However, there is research on the effects of fatigue on muscle function and performance. For example, one study found that muscle fatigue induces a reduction and delay in the activation of both the quadriceps and hamstring muscles in response to rapid destabilising perturbations, potentially reducing the stability around the knee [49]. Intense eccentric and concentric exercises led to a significant decrease in strength, but this decrease rarely surpassed 60% of the original strength, indicating a degree of strength maintenance. In the case of upper-body muscles, the decline in strength at the conclusion of intense eccentric (31.4 ± 20.4%) and concentric (33.6 ± 17.5%) exercises was comparable. However, the strength decline for lower-body muscles was less pronounced following intense eccentric (13.3 ± 12.2%) as opposed to concentric (39.7 ± 13.3%) exercises. The muscle structure and the regular use of lower-body muscles likely provide a protective effect against strength loss during intense eccentric exercises. The researchers found evidence based on three studies that more eccentric than concentric repetitions can be completed at equal relative loads. These results indicate that muscle fatigue may manifest differently between maximal eccentric and concentric resistance exercise [=Nuzzo]. The results imply that prescriptions of eccentric resistance exercise for lower-body muscles should account for greater fatigue resilience of these muscles compared to upper-body muscles.

It is important to note that work fatigue in muscles can be influenced by various factors, including the individual’s physical condition, the intensity and duration of the exercise, and the specific type of muscle contraction (e.g., isometric, isotonic, isokinetic). Further, work fatigue is usually assessed indirectly through measures such as time to exhaustion, reduction in force or power, or increases in perceived effort. While isokinetic dynamometry offers precise quantification of muscle strength and performance, its isolated use has limitations, particularly in assessing functional and real-world muscle performance capabilities. This method primarily focuses on single-joint measurements, which may not fully represent the complex and multi-joint actions required in daily activities. Acknowledging this, our research also incorporated functional assessments such as gait analysis and the Timed Up and Go test [50]. These assessments provide a more holistic understanding of the patient’s functional status and their ability to perform daily activities. Research supports this approach, demonstrating that combining isokinetic testing with functional assessments offers a more comprehensive evaluation of muscle function, particularly in clinical settings.

It is important to emphasise additional limitations. Firstly, the sample size, encompassing 94 individuals (45 in the PCC cohort and 49 in the HCC cohort), while adequate for initial exploration, may limit the generalisability of the findings. A larger cohort would enhance the robustness and external validity of the results. Secondly, the demographic profile of the participants, primarily hospitalised patients aged 20–50 years without chronic diseases, introduces a selection bias. This cohort may not be representative of the broader population of COVID-19 survivors, especially those with pre-existing comorbidities or those who were managed in outpatient settings. Thirdly, including the IPAQ or MLTPAQ questionnaires would have allowed a more accurate determination of the physical fitness of the study groups. Furthermore, the exclusion of patients requiring ICU care and mechanical ventilation could mean that the study does not fully capture the spectrum of COVID-19 severity and its impact on muscle performance. In terms of methodology, the study’s cross-sectional design provides a snapshot of the condition but lacks longitudinal data, which would be crucial in understanding the progression and potential recovery of muscle strength post-COVID-19. Additionally, the reliance on retrospective analysis and telephonic recruitment might have influenced participant selection and data integrity. Future studies could benefit from a prospective design and a more inclusive selection criterion to encompass a wider range of COVID-19 severities and demographic characteristics.

In light of these considerations, the authors suggest caution in interpreting the study’s findings, acknowledging that they represent a specific subset of COVID-19 survivors. This study lays the groundwork for further research, emphasising the need for more comprehensive investigations to fully understand the long-term musculoskeletal impacts of COVID-19.

## 5. Conclusions

In conclusion, this study provides compelling evidence of the impact of post-COVID-19 fatigue on muscle performance, highlighting the importance of considering these effects in the rehabilitation and care of individuals recovering from the virus. This study found that there are differences in skeletal muscle strength and power in people who have been hospitalized for symptomatic COVID 19. These findings contribute to the growing body of research demonstrating the persistent and wide-ranging impacts of COVID-19, extending beyond the acute phase of the disease.

## Figures and Tables

**Table 1:** Detailed characteristics of the study participants (mean ± SD) for the Post-COVID Cohort (PCC) and Healthy Control Cohort (HCC).

Characteristics	PCC (n1 = 45)	HCC (n2 = 49)
Age [years]	38.3 ± 6.2	35.8 ± 5.1
Female/male	23/22	24/25
Height [cm]	169.8 ± 7.3	171.2 ± 10.0
BMI [kg/m^2^]	28.7 ± 5.1	27.7 ± 4.9
COVID-19-associated pneumonia in imaging tests	45	N/A
Oxygen therapy during hospitalisation	39	N/A
Comorbidities	9 (high blood pressure—6, hypothyroidism—1, insulin resistance—1, asthma—1)	N/A
Number of hospitalisation days	(4–12)	N/A
Time interval between the end of hospitalisation and the conduct of the study in weeks	(8–26)	N/A
Symptoms reported by patients concerning COVID-19 - fatigue - muscle pain - joint pain	35 23 15	N/A

Abbreviations: PCC, Post-COVID Cohort; HCC, Healthy Control Cohort; N/A, not applicable.

**Table 2:** Results of a samples *t*-test between Post-COVID Cohort (PCC) and Healthy Control Cohort (HCC) reporting sample sizes (n1, n2).

Variable (Unit)	PCC (n1 = 45)	HCC (n2 = 49)	*t*	*p*	95% CI Lower, Upper	Effect Size (Cohen’s d)
PKTQ_BW.Ext (%)	215.9 ± 21.3	253.8 ± 14.5	−9.9	<0.001	−45.5, −30.3	2.1
PKTQ_BW.Flx (%)	93.2 ± 13.2	109.9 ± 11.9	−6.3	<0.001	−22.0, −11.5	1.3
TIME2PKTQ.Ext (ms)	672.7 ± 54.6	638.9 ± 80.8	2.3	0.02	4.9, 62.7	0.5
TIME2PKTQ.Flx (ms)	696.0 ± 100.3	683.8 ± 148.4	0.5	0.65	−40.9, 65.2	0.1
ANGLE_PKTQ.Ext (°)	67.8 ± 4.0	72.1 ± 5.5	−4.3	<0.001	−6.4, −2.4	0.9
ANGLE_PKTQ.Flx (°)	62.4 ± 6.0	49.3 ± 11.4	6.8	<0.001	9.3, 16.9	1.4
TQ_30DEG.Ext (Nm)	89.8 ± 24.7	105.1 ± 19.5	−3.2	<0.001	−24.5, −5.9	0.7
TQ@30DEG.Flx (Nm)	40.3 ± 13.8	51.0 ± 9.5	−4.3	<0.001	−15.7, −5.8	0.9
COV.Ext (%)	26.2 ± 8.1	17.3 ± 4.1	6.6	<0.001	6.2, 11.6	1.4
COV.Flx (%)	24.2 ± 8.9	21.9 ± 5.6	1.5	0.14	−0.8, 5.4	0.3
WRK_BW.Ext (%)	233.1 ± 24.6	263.5 ± 23.6	−6.0	<0.001	−40.4, −20.3	1.3
WRK_BW.Flx (%)	102.4 ± 15.1	127.6 ± 14.1	−8.2	<0.001	−31.3, −19.1	1.7
WORK1THIRD.Ext (J)	1608.9 ± 349.9	1883.3 ± 322.3	−3.9	<0.001	−415.3, −133.4	0.8
WORK1THIRD.Flx (J)	738.2 ± 154.8	932.7 ± 172.5	−5.6	<0.001	−263.1, −125.8	1.2
WORK3THIRD.Ext (J)	1175.3 ± 186.9	1599.5 ± 308.8	−7.9	<0.001	−531.1, −317.2	1.7
WORK3THIRD.Flx (J)	472.7 ± 72.7	676.7 ± 144.3	−8.5	<0.001	−251.9, −156.1	1.8
WORK FATIGUE.E (%)	37.7 ± 21.1	18.9 ± 13	5.1	<0.001	11.5, 26.1	1.1
WORK FATIGUE.F (%)	56.8 ± 23.5	39.0 ± 11.8	4.6	<0.001	10.1, 25.6	1.0
POWER.Ext (W)	98.4 ± 12.0	103.9 ± 8.3	−2.5	0.01	−9.8, −1.1	0.5
POWER.Flx (W)	39.9 ± 9.2	50.2 ± 8.4	−5.5	<0.001	−14.0, −6.6	1.2
AGON/ANTAG (%)	43.1 ± 3.7	43.4 ± 5.0	−0.3	0.76	−2.1, 1.6	0.1

Note: means ± standard deviation, *t*-statistic, *p*-value, 95% confidence interval around the difference of means and Cohen’s d assessing an effect size for extensors (Ext) and flexors (Flx) of the knee. The test degree of freedom (df) was 92.

## Data Availability

The authors confirm that all data underlying the findings described in this manuscript are fully available to all interested researchers upon request.
